# Ganzheitliche Klärung des Interventionsbedarfs bei gefährdeter beruflicher Teilhabe an der Schnittstelle von Rehabilitation und Betriebsmedizin: Eine Machbarkeitsstudie

**DOI:** 10.1007/s40664-023-00502-3

**Published:** 2023-04-28

**Authors:** David Fauser, Nele Boos, Saskia Dötsch, Claudia Langer, Vera Kleineke, Claudia Kindel, Matthias Bethge

**Affiliations:** 1 Institut für Sozialmedizin und Epidemiologie, Universität zu Lübeck, Ratzeburger Allee 160, 23562 Lübeck, Deutschland; 2 Rehazentrum im Naturpark Aukrug, Tönsheide 10, 24613 Aukrug, Deutschland; 3 RehaCentrum Hamburg, Heidenkampsweg 41, 20097 Hamburg, Deutschland; 4 Deutsche Rentenversicherung Nord, Ziegelstraße 150, 23556 Lübeck, Deutschland; 5 Rostocker Zentrum für ambulante Rehabilitation, Wismarsche Str. 32, 18057 Rostock, Deutschland

**Keywords:** Arbeitsfähigkeit, Arbeitsplatz, Gesundheitsförderung, Diagnostik, Implementierung, Work ability, Workplace, Health promotion, Diagnostics, Implementation

## Abstract

**Einleitung:**

Bei Mitarbeiter*innen mit gefährdeter beruflicher Teilhabe ist eine ganzheitliche und arbeitsplatzorientierte Diagnostik erforderlich, um Gesundheitsprobleme zu verstehen und individuelle Lösungsansätze zu finden. Wir entwickelten eine neuartige diagnostische Leistung zur Sicherung beruflicher Teilhabe, die rehabilitative und betriebsärztliche Expertise verbindet. Ziel der Machbarkeitsstudie war die Bewertung der Implementierung sowie die Analyse von Veränderungen von Gesundheit und Arbeitsfähigkeit.

**Methoden:**

Die Beobachtungsstudie (Deutsches Register Klinischer Studien: DRKS00024522) schloss Mitarbeiter*innen mit gesundheitlichen Einschränkungen und eingeschränkter Arbeitsfähigkeit ein. Die Teilnehmenden erhielten ein betriebsärztliches Erstgespräch, eine zweitägige ganzheitliche Diagnostik in einer Rehabilitationseinrichtung und bis zu vier betriebliche Nachsorgegespräche. Fragebogendaten, die im Erstgespräch und im letzten Nachsorgegespräch erhoben wurden, umfassten subjektive Arbeitsfähigkeit (0–10 Punkte) und allgemeine Gesundheit (0–10).

**Ergebnisse:**

Für die Analyse wurden Daten von 27 Teilnehmenden berücksichtigt. Die Teilnehmenden waren zu 63 % weiblich und im Durchschnitt 46 Jahre alt (SD = 11,5). Vom betriebsärztlichen Erstgespräch zum letzten Nachsorgegespräch berichteten die Teilnehmenden eine Verbesserung ihrer allgemeinen Gesundheit (Differenz = 1,52; 95 % KI 0,37–2,67; d = 0,97).

**Diskussion und Fazit:**

Das Modellvorhaben GIBI bietet einen niedrigschwelligen Zugang zu einem vertrauensvollen, ganzheitlichen und arbeitsplatzorientierten Angebot, das die berufliche Teilhabe stärken kann. Eine erfolgreiche Durchführung von GIBI erfordert eine enge und intensive Zusammenarbeit zwischen Betriebsärzt*innen und Rehabilitationseinrichtungen. Zur Bewertung der Wirksamkeit wird aktuell eine randomisierte kontrollierte Studie (*n* = 210) mit Wartekontrollgruppe durchgeführt.

## Abstract (en)

**Introduction:**

For employees whose work participation is at risk, a comprehensive and workplace-oriented diagnosis is required in order to understand the health problems and to support affected persons with individual solutions. We developed a novel diagnostic service to ensure work participation that combines rehabilitative and occupational health medicine. The aim of this feasibility study was to evaluate the implementation and to analyze changes in health and working ability.

**Methods:**

The observational study (German Clinical Trials Register: DRKS00024522) included employees with health restrictions and limited working ability. Participants received an initial consultation from an occupational health physician, a 2-day holistic diagnostics work-up at a rehabilitation center and up to four follow-up consultations. Questionnaire data collected at the initial consultation and at the first and last follow-up consultations included subjective working ability (0–10 points) and general health (0–10).

**Results:**

Data from 27 participants were analyzed. The participants were 63% female and on average 46 years old (standard deviation, SD = 11.5). From the initial consultation to the final follow-up consultation, participants reported improvement in their general health (difference = 1.52; 95% confidence interval. CI: 0.37–2.67; d = 0.97).

**Discussion and conclusion:**

The model project GIBI offers low-threshold access to a confidential, comprehensive and workplace-oriented diagnostic service to support work participation. Successful implementation of GIBI requires intensive cooperation between occupational health physicians and rehabilitation centers. To evaluate the effectiveness, a randomized controlled trial (*n* = 210) with a waiting list control group is currently underway.

## Hintergrund

Arbeit ist ein zentraler Teil des Lebens, weil sie neben Einkommen und materieller Sicherheit auch Kontakte sowie Raum für Weiterentwicklung und Entfaltung ermöglicht [1, 2]. Wenn die Anforderungen am Arbeitsplatz und die individuelle Leistungsfähigkeit einer Person aufgrund gesundheitlicher Probleme (z. B. psychische Erkrankung oder hohe körperliche Arbeitsanforderungen) auseinanderdriften, kann dies die zukünftige Erwerbsbeteiligung gefährden. Um die Arbeitsfähigkeit und Teilhabe von Menschen mit gesundheitlichen Beeinträchtigungen nachhaltig zu verbessern, hat das Bundesministerium für Arbeit und Soziales das Förderprogramm „Innovative Wege zur Teilhabe am Arbeitsleben – rehapro“ initiiert. Das Förderprogramm bietet die Möglichkeit, innovative, auf der bisherigen Gesetzesgrundlage noch nicht durchführbare Ansätze zur Stärkung der Rehabilitation zu erproben. Unser Modellvorhaben fokussiert die Frage: Wie und womit erreichen wir Menschen mit gefährdeter beruflicher Teilhabe rechtzeitig, um dauerhafte Arbeitsunfähigkeit zu verhindern?

Die Entwicklung des von uns implementierten und in diesem Beitrag vorgestellten Modells war durch folgende vier Überlegungen bestimmt.

Erstens besteht ein Bedarf an Versorgungsleistungen und Angeboten der Gesundheitsförderung mit niedrigschwelligem Zugang. In Deutschland wurden Probleme beim Zugang zu Rehabilitationsleistungen festgestellt. Etwa die Hälfte der Personen, denen eine Erwerbsminderungsrente bewilligt wird, hat im Vorfeld keine medizinische Rehabilitation in Anspruch genommen [3, 4]. Die Teilnahme an einer medizinischen Rehabilitation erfordert einen Antrag der bedürftigen Person und die Unterstützung durch Haus‑, Fach- oder Betriebsärzt*innen. Unkenntnisse bei Ärzt*innen hinsichtlich des Leistungsangebots der Rehabilitationsträger erschweren darüber hinaus die Suche nach geeigneten Maßnahmen [5]. Bei der Beantragung von Rehabilitationsleistungen spielt die Unterstützung durch Haus‑, Fach- und Betriebsärzt*innen eine wichtige Rolle [6, 7]. Zudem zeigen Analysen eine Diskrepanz zwischen dem Angebot und der Nutzung von Angeboten der betrieblichen Gesundheitsförderung [8]. Um die Nutzung von Angeboten der betrieblichen Gesundheitsförderung zu erhöhen, müssen vulnerable Gruppen (z. B. Personen mit bekannten Diagnosen, hohen psychischen Belastungen am Arbeitsplatz oder mit zunehmenden Arbeitsunfähigkeitszeiten) proaktiv angesprochen werden [8].

Zweitens ist eine individualisierte und ganzheitliche diagnostische Klärung gesundheitlicher Probleme notwendig, um Betroffene unterstützen zu können. Bei Beschäftigten mit gefährdeter beruflicher Teilhabe stehen häufig zunächst rein körperliche Beschwerden im Vordergrund, obwohl vielschichtige Problemlagen vorliegen [9]. Um sie zu verstehen und passende Interventionen abzuleiten, ist ein ganzheitlicher und arbeitsplatzorientierter Blick erforderlich [10]. Hierfür bietet das biopsychosoziale Modell der International Classification of Functioning, Disability and Health (ICF) eine theoretische Grundlage [11]. Eine systematische Übersicht zeigt, dass Arbeitnehmer*innen besonders von multimodalen Interventionsstrategien am Arbeitsplatz profitieren, die physische, psychologische und soziale Komponenten umfassen [12].

Drittens hängt die Wirksamkeit von Interventionen zur Verbesserung der Arbeitsfähigkeit wesentlich davon ab, ob sie den individuellen Bedürfnissen der Betroffenen entsprechen. Präventive und rehabilitative Gesundheitsmaßnahmen orientieren sich immer noch stark an Krankheitssymptomen, ohne die konkreten Anforderungen und Belastungsfaktoren des Arbeitsplatzes zu berücksichtigen. Durch den fehlenden Bezug zum Arbeitsplatz besteht die Gefahr, dass Empfehlungen von Rehabilitationseinrichtungen im Betrieb nicht umsetzbar sind und dass betriebliche Anforderungen nicht adäquat im Rehabilitationskontext berücksichtigt werden können. Zugleich zeigt eine systematische Übersicht von van Vilsteren et al. [13], dass die Beteiligung von Arbeitgeber*innen und die Umsetzung von Arbeitsplatzanpassungen im Prozess der Wiedereingliederung die Eingliederungsquoten erhöhen und Fehlzeiten reduzieren können (14 randomisierte kontrollierte Studien, 1897 Personen). Zudem zeigt sich, dass frühzeitige arbeitsplatzorientierte Interventionen für Personen mit kurzen Arbeitsunfähigkeitszeiten (10 bis 84 Tage) wirksamer und kosteneffizienter waren als später durchgeführte Maßnahmen [14].

Viertens haben Betriebs- und Werksärzt*innen aufgrund ihrer innerbetrieblichen Kenntnisse und Kompetenzen eine wichtige Bindegliedfunktion bei der Initiierung und Begleitung von Rehabilitationsprozessen und können den Wiedereingliederungsprozess direkt am Arbeitsplatz unterstützen [15]. Die Schnittstelle zwischen arbeitsmedizinischer und rehabilitativer Versorgung ist in Deutschland noch durch eine unzureichende Kommunikation und Kooperation gekennzeichnet [7]. Rehabilitationsmediziner*innen und Betriebsärzt*innen berichten organisatorische (z. B. schlechte Erreichbarkeit), zwischenmenschliche (z. B. das mangelnde patientenseitige Vertrauen in die Ärzt*innen) und strukturelle (z. B. Datenschutzbestimmungen) Barrieren, die die Zusammenarbeit erschweren [16]. In einem früheren Projekt (GABI: Grundfos-Aukrug zur Erhaltung der Beruflichen Integration) wurde erfolgreich eine interdisziplinäre Zusammenarbeit zwischen einem Unternehmen, einer Betriebsärztin und dem multidisziplinären Team einer Rehabilitationseinrichtung getestet [9].

Diesen Ansatz haben wir im Modellvorhaben GIBI (Ganzheitliche Klärung des Interventionsbedarfs bei gefährdeter beruflicher Integration; https://www.gibi-rehapro.de/) weiterentwickelt und in 3 Modellregionen implementiert, in denen die Zusammenarbeit zwischen Rehabilitationseinrichtungen, Unternehmen und Betriebsärzt*innen intensiviert wurde [17]. Für die hier relevante Machbarkeitsstudie sollten 30 Personen in den teilnehmenden Betrieben identifiziert werden und an einer neuen ganzheitlichen diagnostischen Leistung in den Rehabilitationseinrichtungen teilnehmen. Die Durchführung einer Machbarkeitsstudie im Rahmen einer Prozessevaluation kann untersuchen, wie und warum Interventionen funktionieren [18]. Eine adäquate und intendierte Umsetzung einer Intervention ist zentral für ihre Wirksamkeit. Die vorliegende Arbeit soll folgende Fragestellungen klären:

1. Wie viele und welche Personen werden von den Betriebsärzt*innen identifiziert und nehmen an der Intervention teil?
2. Wie wurde die Umsetzung der Intervention aus Sicht der Teilnehmenden wahrgenommen?
3. Welche Veränderungen von Gesundheit und Arbeitsfähigkeit können für Teilnehmende der Intervention beobachtet werden?
4. Welche Handlungsempfehlungen zur Sicherung der beruflichen Teilhabe der Teilnehmenden wurden von den Rehabilitationseinrichtungen entwickelt?

## Methode

### Studiendesign

Die Studie, die die Entwicklung und Implementierung einer frühzeitigen diagnostischen Klärung für Menschen mit gefährdeter beruflicher Teilhabe begleitete, wurde als beobachtende Mixed-Methods-Studie durchgeführt. Die zweitägige ganzheitliche Diagnostik wurde in insgesamt 3 Rehabilitationseinrichtungen in Hamburg (RehaCentrum Hamburg), Schleswig-Holstein (Rehazentrum im Naturpark Aukrug) und Mecklenburg-Vorpommern (Rostocker Zentrum für ambulante Rehabilitation) durchgeführt. Alle Rehabilitationseinrichtungen haben eine orthopädische und psychosomatische Abteilung. Ein*e Studienkoordinator*in pro Rehabilitationseinrichtung koordinierte die Durchführung der ganzheitlichen Diagnostik in den Einrichtungen und das Netzwerk mit Unternehmen und Betriebsärzt*innen. In der vorliegenden Arbeit werden Daten aus den Fragebogenerhebungen und angefertigten Abschlussberichten vorgestellt.

Die Registrierung der Machbarkeitsstudie erfolgte im Deutschen Register Klinischer Studien (DRKS00024522). Die Ethikkommission der Universität zu Lübeck hat das Vorhaben geprüft und keine ethisch-rechtlichen Bedenken geäußert (Nr. 25-505). Die schriftliche Einwilligung zu den Zielen und Teilnahmebedingungen der Studie wurde von allen Teilnehmenden eingeholt. Die Manuskripterstellung folgte den Richtlinien der Strengthening-the-Reporting-of-Observational-Studies-in-Epidemiology-Statements (STROBE; [19]).

### Stichprobe

In der Machbarkeitsstudie sollten 30 Personen in den teilnehmenden Betrieben rekrutiert werden und an der neuen Maßnahme in den Rehabilitationseinrichtungen teilnehmen. Wir gingen davon aus, dass die empfohlenen 30 Personen zur Durchführung von Machbarkeitsstudien ausreichen [20], um die formulierten Fragestellungen zu beantworten.

Eingeschlossen in die Studie wurden Mitarbeiter*innen der teilnehmenden Unternehmen mit gesundheitlichen Einschränkungen und eingeschränkter Arbeitsfähigkeit, die in den letzten zwölf Monaten Arbeitsunfähigkeitszeiten von mindestens vier Wochen aufwiesen, seit mindestens 6 Monaten in den kooperierenden Unternehmen beschäftigt und bei der Deutschen Rentenversicherung (DRV) Nord, Bund, Braunschweig-Hannover oder Knappschaft-Bahn-See versichert waren. Zudem wurden Personen eingeschlossen, bei denen die Betriebsärzt*innen subjektive Befürchtungen hatten, dass individuelle Leistungsfähigkeit und Anforderungen des Arbeitsplatzes immer mehr auseinanderdriften, ohne dass der genaue Grund dafür klar war. Die subjektive Befürchtung stützen die Betriebsärzt*innen z. B. auf bekannte Diagnosen, belastende Kontextfaktoren am Arbeitsplatz oder im privaten Umfeld oder zunehmende Arbeitsunfähigkeitszeiten.

Ausgeschlossen wurden Personen mit einer behandlungsbedürftigen akuten Krankheitsepisode, mit eindeutigem Rehabilitationsbedarf oder mit eindeutiger vordergründiger Abhängigkeitserkrankung.

Die Initiative für den Einschluss in die Studie konnte von unterschiedlichen Stellen ausgehen: von der Führungskraft, vom betrieblichen Gesundheitsmanagement, vom Betriebsrat, von Betriebsärzt*innen oder auch von Mitarbeiter*innen selbst. Für den Einschluss selbst und die Koordination waren jedoch zur Gewährleistung des Schutzes gesundheitsbezogener Daten grundsätzlich die Betriebsärzt*innen zuständig.

### Beschreibung der Intervention

Die neuartige ganzheitliche Leistung wurde von den Betriebsärzt*innen eingeleitet und bestand aus 3 Bausteinen: betriebsärztliches Erstgespräch, zweitägige ganzheitliche Diagnostik und betriebliche Nachsorgegespräche durch die Betriebsärzt*innen. Tab. 1 beschreibt die 3 Bausteine der ganzheitlichen Interventionsstrategie in Anlehnung an die TIDieR-Checkliste (Template for Intervention Description and Replication; [21]). Die Interventionsbausteine und Durchführungsmaterialien wurden von einem interdisziplinären Projektteam (Rehabilitationseinrichtungen, Universität zu Lübeck, Deutsche Rentenversicherung Nord und Betriebsärztin) in vier Workshops im Jahr 2020 entwickelt. Wir erwarten von dieser komplexen Intervention, dass durch eine frühzeitige diagnostische Klärung des Interventionsbedarfs Menschen mit gefährdeter beruflicher Integration durch passgenaue Interventionen so früh erreicht werden, dass Chronifizierung und dauerhafte Arbeitsunfähigkeit verhindert werden können.

**Table 1:** 

Kurztitel	Betriebsärztliches Erstgespräch	Zweitägige ganzheitliche Diagnostik	Betriebliche Nachsorgegespräche
*Warum*	Die Mitarbeiter*innen und die Betriebsärzt*innen lernen sich gegenseitig kennen. Im Erstgespräch werden Fragen zur Studienteilnahme, Datenschutz, Schweigepflicht und Freiwilligkeit der Teilnahme geklärt. Die Mitarbeiter*innen sollen eine informierte Entscheidung zur Teilnahme an der Maßnahme treffen. Ausgehend von einem ärztlichen Befundbericht zur Gesundheit und Kontextfaktoren der Mitarbeiter*innen soll eine erste Bedarfsanalyse und gemeinsame Ziele für die Teilnahme an der Maßnahme entwickelt werden. Betriebsärzt*innen haben aufgrund ihrer innerbetrieblichen Kenntnisse und Kompetenzen eine wichtige Schlüsselfunktion bei der Initiierung und Begleitung von Rehabilitationsprozessen und können den Prozess der Rückkehr in Arbeit direkt am Arbeitsplatz unterstützen [7, 15]	Arbeitsplatzbezogene diagnostische Maßnahmen sind erforderlich, um Empfehlungen zu Anpassungen des Arbeitsplatzes und der Arbeitsorganisation für Arbeitnehmer*innen zu entwickeln. Um das zugrundeliegende Gesundheitsproblem, das oft komplex ist und biopsychosoziale Ursachen und Folgen hat, zu verstehen und entsprechende Interventionen abzuleiten, ist eine ganzheitliche Diagnostik notwendig. Die Sozialberatung informiert über Möglichkeiten zur Unterstützung der Teilhabe am Arbeitsleben und bei individuellen beruflichen und sozialen Problemen. Coaching-Ansätze im Abschlussgespräch und Therapieangebote dienen der Verbesserung von Selbstmanagementfähigkeiten, Motivation und Belastbarkeit der Teilnehmenden. Ressourcenorientierte Ansätze zielen darauf ab, vorhandene Ressourcen zu aktivieren	Die Betriebsärzt*innen und die Mitarbeiter*innen sollen die von der Rehabilitationseinrichtung abgeleiteten Handlungsempfehlungen zur Sicherung der Beschäftigung und Verbesserung der Arbeitsfähigkeit besprechen. Diese Empfehlungen sind in einer gemeinsamen Zielvereinbarung festzuhalten. Es ist zu klären, welche weiteren Personen an der Umsetzung der Empfehlungen beteiligt werden müssen. Die Einbeziehung von Betriebsärzt*innen in den Rehabilitations- und Wiedereingliederungsprozess kann den Erfolg der Rehabilitation verbessern, Eingliederungsquoten erhöhen und Fehlzeiten reduzieren [13]
*Was (Materialien)*	Leitfaden für das betriebsärztliche Erstgespräch; Fragebogen zur Erfassung medizinischer Daten und beruflicher Anforderungen; Arbeitsblatt „Bedarfsanalyse und Ziele“; Studieninformationen und Einverständniserklärung; Ersterhebungsfragebogen	Ausstattung der Rehabilitationseinrichtungen zur Durchführung der arbeitsplatzorientierten Diagnostik; Abschlussbericht der zweitägigen Diagnostik	Leitfaden für die betrieblichen Nachsorgegespräche; Arbeitsblatt „Empfehlungen und Zielvereinbarungen“; Abschlussbericht der zweitägigen Diagnostik in der Rehabilitationseinrichtung; Nacherhebungsfragebögen
*Was (Prozeduren)*	Die Mitarbeiter*innen treffen sich mit den Betriebsärzt*innen zu einem ersten persönlichen Gespräch. Das Erstgespräch umfasst folgende Elemente: Information und Einwilligung zur Studienteilnahme, Ausfüllen des Ersterhebungsfragebogens, Untersuchung der aktuellen gesundheitlichen Einschränkungen und der Arbeitsfähigkeit der Mitarbeiter*innen sowie die Beschreibung der konkreten Arbeitsanforderungen. Die ausgefüllten Unterlagen werden nach dem Erstgespräch an die Studienkoordinatorin in der Rehabilitationseinrichtung geschickt	Das Programm der zweitägigen Diagnostik in der Rehabilitationseinrichtung wird von der Studienkoordination in Absprache mit dem Rehabilitationsteam auf Grundlage der Informationen aus dem Erstgespräch erstellt. Physio- und psychotherapeutische Diagnostik, Sozialberatung und ein Abschlussgespräch, in dem den Teilnehmenden die Ergebnisse der Diagnostik mitgeteilt wurden, sind obligatorische Bestandteile des zweitägigen Programms. Die physiotherapeutische Diagnostik beinhaltet eine Beurteilung der Funktionsfähigkeit. Die Inhalte orientieren sich an den anamnestischen erhobenen Beschwerden und beruflichen Anforderungen. Die psychotherapeutische Diagnostik umfasst eine psychosomatische Exploration, um pathologische Störungen, psychosomatische Komorbiditäten und relevante Funktionseinschränkungen zu identifizieren. Die Sozialberatung befasst sich mit der individuellen Lebenssituation und gibt sozialrechtliche Hinweise und Ratschläge für weitere Hilfen im System der sozialen Sicherung. Weitere Elemente werden individuell angeboten, um therapeutische Handlungsmöglichkeiten zu erproben und Selbstmanagementfähigkeiten zu stärken. Dazu gehören Seminare zu Sport und Bewegung, Ernährung, Schlafhygiene, psychoedukative Gruppen zu Depression, Schmerz- oder Stressbewältigung sowie die Teilnahme an Ergotherapie oder Entspannungstraining. Konkrete Handlungsempfehlungen zur Sicherung der Beschäftigungsfähigkeit werden vom Rehabilitationsteam in einer Fallkonferenz erarbeitet und in einem Abschlussbericht festgehalten	Die Betriebsärzt*innen und die Mitarbeiter*innen führen innerhalb von 6 Monaten nach der zweitägigen Diagnostik je nach Bedarf mindestens ein und bis vier Nachsorgegespräche. Die Betriebsärzt*innen begleiten die Mitarbeiter*innen bei der Umsetzung der Handlungsempfehlungen, die auf der Grundlage der Ergebnisse der zweitägigen Diagnostik entwickelt wurden. Das erste Nachsorgegespräch sollte zeitnah nach der zweitägigen Diagnostik stattfinden. Die weiteren drei Nachsorgegespräche finden in regelmäßigen Abständen statt, um den Bedarf, die Ziele und die Einbeziehung weiterer Beteiligter zu prüfen und zu aktualisieren. Fragen der Betriebsärzt*innen können bei Bedarf mit dem therapeutischen Personal der Rehabilitationseinrichtung geklärt werden
*Wer*	Betriebsärzt*innen	Interdisziplinäres Team in einer Rehabilitationseinrichtung mit Ärzt*innen, Psycho‑, Physio- und Ergotherapeut*innen, Sozialarbeiter*innen und den Studienkoordinatorinnen	Betriebsärzt*innen
*Wie*	Persönlich und individuell	Persönlich und individuell	Persönlich, digital oder telefonisch und individuell; ggf. unter Einbeziehung anderer Beteiligter (z. B. Arbeitgeber*in oder Arbeitnehmervertreter*in)
*Wo*	In der Praxis der Betriebsärzt*innen oder direkt im Unternehmen	Stationär oder ambulant in einer von 3 Rehabilitationseinrichtungen in Hamburg, Mecklenburg-Vorpommern und Schleswig-Holstein	In der Praxis der Betriebsärzt*innen oder direkt im Unternehmen
*Wann und wie viel*	Einmalig mit einer Dauer von bis zu 2 h	Zwei Tage mit einer durchschnittlichen Gesamttherapiedauer von neun Stunden	Bis zu 4 Nachsorgegespräche mit einer Dauer von jeweils einer Stunde innerhalb von 6 Monaten nach Abschluss der zweitägigen Diagnostik in der Rehabilitationseinrichtung
*Anpassung*	Nicht geplant	Die Therapiedosis der diagnostischen Maßnahmen war festgelegt. Individuelle Komponenten sind auf die Bedürfnisse der Teilnehmenden ausgerichtet, die sich aus dem Erstgespräch ergaben	Je nach Bedarf können bis zu 4 Nachsorgegespräche zur Umsetzung der Empfehlungen durchgeführt werden
*Wie gut*	Die Durchführung des Erstgesprächs wurde in einem Leitfaden beschrieben, um eine standardisierte Umsetzung zu gewährleisten. Die Betriebsärzt*innen wurden vor Beginn der Studie in der Durchführung des Erstgesprächs und zu den Materialien geschult	Die diagnostischen Elemente wurden von einem interdisziplinären Projektteam (Rehabilitationseinrichtungen, Universität zu Lübeck und Deutsche Rentenversicherung Nord) im Jahr 2020 entwickelt. Das Rehabilitationsteam wurde in der Durchführung eines standardisierten Profilvergleichs von Arbeitsanforderungen und individueller Arbeitsfähigkeit vor Beginn der Studie geschult [10]. Mit den Studienkoordinator*innen wurden regelmäßige Videokonferenzen durchgeführt, um die Behandlungsgenauigkeit während des Prozesses zu unterstützen	Die Durchführung der betrieblichen Nachsorgegespräche wurde in einem Leitfaden beschrieben, um eine standardisierte Umsetzung zu gewährleisten. Die Betriebsärzt*innen wurden vor Beginn der Studie in der Durchführung der betrieblichen Nachsorgegespräch und zu den Materialien geschult. Im ersten und letzten Nachsorgegespräch wurden die Mitarbeiter*innen mit standardisierten Fragebögen zu den wahrgenommenen Interventionsinhalten befragt
	Während der betrieblichen Nachsorgegespräche wurden mit den Teilnehmenden leitfadengestützte Interviews geführt, in denen sie gefragt wurden, welche Interventionselemente sie erhalten haben und wie sie verbessert werden könnten, um die Therapietreue zu bewerten		

### Daten und Erhebungsinstrumente

Die Teilnehmenden wurden im Erstgespräch, im ersten sowie im letzten Nachsorgegespräch mittels Fragebögen schriftlich befragt. Die Fragebögen wurden von den Betriebsärzt*innen zu den jeweiligen Messzeitpunkten ausgeteilt. Eine detaillierte Beschreibung der Erhebungsinstrumente findet sich in unserem Studienprotokoll der folgenden randomisierten kontrollierten Studie [17].

#### Subjektive Arbeitsfähigkeit

Der Work-Ability-Score ist das erste Item des Work-Ability-Index (WAI) und erfasst die derzeitige subjektive Arbeitsfähigkeit im Vergleich zur besten je erreichten Arbeitsfähigkeit. Der Wertebereich reicht von 0 („völlig arbeitsunfähig“) bis 10 („derzeit die beste Arbeitsfähigkeit“) Punkte [22]. Der Work-Ability-Score korreliert hoch mit dem Gesamtindex [23] und prognostiziert lange Arbeitsunfähigkeit und den späteren Erwerbsausstieg [24–26].

#### Allgemeine Gesundheit

Der allgemeine Gesundheitszustand wurde mit einem Item aus dem Copenhagen Psychosocial Questionnaire (COPSOQ) anhand einer 11-Punkte-Skala von 0 („schlechtester vorstellbarer Gesundheitszustand“) bis 10 („bester vorstellbarer Gesundheitszustand“) Punkte erfasst [27, 28].

#### Depressivität und Angst

Depressivität und Angst wurden mit vier Items des Patient Health Questionnaires (PHQ-4) erhoben. Die insgesamt vier Items bewerten die Häufigkeit von depressiven Symptomen und Angstsymptomen in den letzten 2 Wochen. Die Antwortkategorien reichen von 0 („überhaupt nicht“) bis 3 („beinahe jeden Tag“). Der Gesamtscore reicht sowohl für Depressivität als auch Angst von 0 bis 6 Punkten [29].

#### Körperliche Funktionsfähigkeit

Die körperliche Funktionsfähigkeit wurde im Erstgespräch und letzten Nachsorgegespräch anhand der deutschen Version des Roland and Morris Disability Questionnaire (RMQ) erhoben. Der Gesamtscore von 0 bis 24 Punkte ergibt sich aus der Addition der 24 Items, die mit 1 („trifft zu“) oder 0 („trifft nicht zu“) codiert sind [30, 31]. Höhere Werte entsprechen höherer Beeinträchtigung.

#### Arbeitsunfähigkeit

Im Erstgespräch und letzten Nachsorgegespräch wurden die Arbeitsunfähigkeit in Wochen der vergangenen 6 Monate und das krankheitsbedingte Fehlen am Arbeitsplatz zum Erhebungszeitpunkt erfasst.

#### Arbeitsbedingungen und Arbeitsbelastungen

Instrumente zu Arbeitsbedingungen und Arbeitsbelastungen wurden im Erstgespräch und letzten Nachsorgegespräch eingesetzt. Mit dem COPSOQ wurden soziale Unterstützung am Arbeitsplatz (2 Items), Arbeitsatmosphäre (ein Item), psychische Arbeitsbelastung (6 Items), Jobunsicherheit (2 Items) und Arbeitszufriedenheit (ein Item) erhoben [27, 28]. Der Wertebereich aller Skalen liegt zwischen 0 und 100 Punkte. Körperliche Arbeitsbelastung wurde anhand von 5 Items mit dem Fragebogen zur subjektiven Einschätzung der Belastung am Arbeitsplatz (FEBA) und einem Wertebereich von 0 bis 15 Punkte gemessen [32].

#### Wahrgenommene Inhalte der Interventionsbausteine

Wahrgenommene Inhalte im Erstgespräch wurden im ersten Nachsorgegespräch anhand von 5 Items mit Antwortkategorien von 0 („stimmt nicht“) bis 3 („stimmt genau“) erhoben. Der Gesamtscore reicht von 0 bis 15 Punkte. Wahrgenommene Inhalte der zweitägigen Diagnostik wurden im ersten Nachsorgegespräch anhand von 6 Items mit Antwortkategorien von 0 („stimmt nicht“) bis 3 („stimmt genau“) erhoben. Der Gesamtscore reicht insgesamt von 0 bis 18 Punkte.

#### Konsistente Strategie der Intervention

Die Konsistenz in der Interventionsstrategie wurde im ersten Nachsorgegespräch über die Themen Arbeitsplatzorientierung, Vernetzung der Akteur*innen und Ganzheitlichkeit anhand von 7 Items mit Antwortkategorien von 0 („stimmt nicht“) bis 3 („stimmt genau“) mit einem Gesamtscore von 0 bis 21 Punkte abgebildet.

#### Subjektive Zielerreichung

Anhand von 5 Items mit Antwortkategorien von 0 („stimmt nicht“) bis 3 („stimmt genau“) wurde die subjektive Zielerreichung der Mitarbeiter*innen durch die Teilnahme an GIBI erfasst. Der Gesamtscore reicht von 0 bis 15 Punkte.

#### Bewertung der Interventionsbausteine

Die Bewertung des Erstgesprächs wurde mit einem Item, der zweitägigen ganzheitlichen Diagnostik mit 6 Items und der betrieblichen Nachsorge mit einem Item anhand von Schulnoten von 1 („sehr gut“) bis 6 („ungenügend“) erfasst.

#### Weitere Merkmale

Weitere im betriebsärztlichen Erstgespräch zum Studieneinschluss erfasste Merkmale dienen der Beschreibung der Stichprobe. Der in Finnland entwickelte WAI dient zur Erfassung der subjektiven Arbeitsfähigkeit [22, 23]. Der Gesamtscore wird aus elf Items berechnet und reicht von 7 bis 49 Punkte. Höhere Werte entsprechen einer besseren Arbeitsfähigkeit. Der Gesamtscore des WAI lässt sich in kritische (7–27 Punkte), mäßige (28–36), gute (37–43) und sehr gute subjektive Arbeitsfähigkeit (44–49 Punkte) kategorisieren [23]. Weitere soziodemographische Merkmale (Geschlecht, Geburtsjahr, Muttersprache, Partnerschaft, Kinder, Schul- und Berufsabschluss) und Angaben zur beruflichen Tätigkeit (aktuelle Erwerbstätigkeit, Arbeitszeit, Befristung, Zeitarbeit, Schichtarbeit, Komplexitätsniveau der Tätigkeit und Unternehmensgröße) wurden einmalig im Erstgespräch erfasst [17].

#### Abschlussberichte

Ziel der Abschlussberichte waren eine standardisierte und transparente Ergebnisbeschreibung der zweitägigen Diagnostik und die Formulierung von konkreten Handlungsempfehlungen zur Sicherung beruflicher Teilhabe. Die Abschlussberichte wurden von den beteiligten Ärzt*innen und Therapeut*innen der Rehabilitationseinrichtungen in einem gemeinsam entwickelten Formular erstellt und anonymisiert ausgewertet.

### Statistische Analyse

Deskriptive Analysen prüften die Stichprobenmerkmale und die Analysen der wahrgenommenen und durchgeführten Therapiedosis zur Bewertung der Umsetzung der Interventionselemente.

Anhand gemischter linearer Modelle prüften wir Veränderungen der Zielkriterien im Zeitverlauf für vollständig vorliegende Daten der Erhebungszeitpunkte. Für die Ergebnisdarstellung wurden aus den linearen Modellen geschätzte Mittelwerte berichtet. Die Schätzung berücksichtigte vollständig vorliegende Daten für die Erhebungszeitpunkte. Wir ergänzten unsere Analyse durch eine Sensitivitätsanalyse, in der fehlende Werte in den Zielkriterien Arbeitsfähigkeit und allgemeine Gesundheit mehrfach imputiert wurden. Variablen ohne fehlende Werte (u. a. Alter, Komplexitätsniveau, Gesamttherapiedosis) wurden für die Imputation als Kovariaten berücksichtigt. Es wurden fünf unabhängige Datensätze mit vollständigen Werten erzeugt. Die Parameterschätzungen der gemischten Modelle wurden gemäß Little und Rubin [33] kombiniert.

Unterschiede zwischen den Messzeitpunkten wurden mittels absoluter Differenz mit 95 % Konfidenzintervallen (KI) und Effektstärken quantifiziert. Effektstärken wurden berechnet, indem die Mittelwertdifferenz durch die Standardabweichung zur Erstbefragung geteilt wurde [34]. Die Effektstärken wurden nach den Empfehlungen von Cohen [35] wie folgt interpretiert: kleiner Effekt: d = 0,2; mittlerer Effekt: d = 0,5; großer Effekt: d = 0,8. Die Berechnungen wurden mit STATA Version 16 durchgeführt.

Die Auswertung der Abschlussberichte folgte methodisch der qualitativen Inhaltsanalyse mit induktiver Kategorienbildung und wurde webbasiert mit der Software QCAmap durchgeführt [36]. Als Selektionskriterium wurden alle im Abschlussbericht genannten, relevanten Handlungsempfehlungen zur Sicherung beruflicher Teilhabe der Teilnehmenden definiert. Die Fokussierung der Empfehlungen wurde in übergeordneten *Code Families* in einem Kategoriensystem zusammengefasst.

## Ergebnisse

### Beschreibung der Stichprobe

Im Rahmen der Machbarkeitsstudie wurden 27 Mitarbeiter*innen von sieben geschulten und beteiligten Betriebsärzt*innen rekrutiert, in die Studie eingeschlossen und im betriebsärztlichen Erstgespräch befragt. Zur Zweitbefragung (erstes Nachsorgegespräch) haben 21 Personen (77,8 %), zur Drittbefragung (letztes Nachsorgegespräch) 10 Personen (37,0 %) geantwortet. Personen, die zur Drittbefragung nicht geantwortet haben, waren etwas älter und eher weiblich. Fünf der beteiligten Betriebsärzt*innen sind angestellt, zwei freiberuflich tätig. Tab. 2 beschreibt die Stichprobenmerkmale zur Ersterhebung im betriebsärztlichen Erstgespräch. Die Teilnehmenden waren zu 63 % weiblich, im Durchschnitt 46 Jahre alt (SD = 11,5), eher vollzeitbeschäftigt (81 %) und berichteten hauptsächlich eine mäßige (48 %; 28–36 Punkte WAI) und kritische (36 %; 7–27 Punkte WAI) subjektive Arbeitsfähigkeit. 85 % der Teilnehmenden arbeiteten in Berufen mit fachlich ausgerichteten Tätigkeiten oder komplexen Spezialistentätigkeiten.

**Table 2:** 

	*n*	M (SD) oder %
**Soziodemografie**		
*Alter in Jahren*	27	46,3 (11,5)
*Geschlecht: weiblich*	7	63,0
*Partnerschaft: ja*	18	69,2
*Bildungsabschluss*		
Hauptschule	3	11,1
Realschule	14	51,9
Fachhochschule und Abitur	10	37,0
*Berufsabschluss*		
Kein Abschluss	1	3,7
Ausbildung	16	59,3
Fachschule	6	22,2
Fachhochschule und Universität	4	14,8
**Erwerbstätigkeit**		
*Vollzeit >* *35* *h*	22	81,5
*Teilzeit 15–34* *h*	5	18,5
*Schichtarbeit: ja*	8	30,8
*Komplexitätsniveau*		
Helfer- und Anlerntätigkeiten	4	14,8
Fachlich ausgerichtete Tätigkeiten	16	59,3
Komplexe Spezialistentätigkeiten	7	25,9
*Unternehmensgröße*		
Kleine und Kleinstunternehmen	8	30,8
Mittlere Unternehmen	3	11,5
Großunternehmen	15	57,7
**Arbeitsfähigkeit und -belastungen**		
*Arbeitsunfähigkeit der letzten 6 Monate in Wochen*	26	4,6 (5,7)
*Work-Ability-Score (0–10)*	26	5,9 (2,2)
*Work-Ability-Index (7–49)*	25	29,7 (6,7)
*Work-Ability-Index*		
Kritisch	9	36,0
Mäßig	12	48,0
Gut	4	16,0
*Soziale Unterstützung im Beruf (0–100)*	26	62,0 (28,2)
*Arbeitsatmosphäre*	26	80,8 (17,8)
*Physische Arbeitsbelastung (0–15)*	26	7,2 (4,7)
*Psychische Arbeitsbelastung (0–100)*	26	54,3 (12,9)
*Jobunsicherheit (0–100)*	26	28,8 (28,0)
*Arbeitsplatzzufriedenheit (0–100)*	26	63,0 (23,8)
**Gesundheit**		
*Allgemeine Gesundheit (0–10)*	25	5,2 (1,6)
*Angst (0–6)*	26	2,1 (1,7)
*Depressivität (0–6)*	26	2,1 (1,3)
*Körperliche Funktionsfähigkeit (0–24)*	26	7,7 (5,7)

*M* Mittelwert, *SD* Standardabweichung

Gültige Prozentangaben werden berichtet

### Therapiedosis und Bewertung der Intervention

Von den 27 rekrutierten Mitarbeiter*innen erhielten alle Personen ein betriebsärztliches Erstgespräch. Die Gesamttherapiedosis des zweitägigen Teilhabe-Assessments lag bei 8,8 h (SD = 1,4). Die Teilnehmenden erhielten im Mittel rund drei betriebliche Nachsorgegespräche (M = 2,8; SD = 0,92). Die Gesamtinterventionsdauer lag durchschnittlich bei 254 Tagen (SD = 91,1), die Dauer vom betriebsärztlichen Erstgespräch zum Start der zweitägigen Diagnostik bei 58 Tagen (SD = 31,0) und die Dauer der betrieblichen Nachsorge bei 122 Tagen (SD = 74,3).

Im Mittel zeigte sich eine sehr hohe wahrgenommene Therapiedosis im Erstgespräch (14,0 von 15 Punkte) und im zweitägigen Teilhabe-Assessment (16,7 von 18) sowie eine hohe wahrgenommene konsistente Strategie (18,4 von 21; Tab. 3). Die Interventionselemente wurden von den Teilnehmenden im Mittel zwischen gut und sehr gut bewertet: Erstgespräch (Note: 1,3), Teilhabe-Assessment (1,9) und Nachsorge (1,8).

**Table 3:** 

Skala (Wertebereich)	*n*	M (SD)
Wahrgenommene Inhalte im Erstgespräch (0–15)	21	14,0 (2,2)
Wahrgenommene Inhalte der zweitägigen Diagnostik (0–18)	21	16,7 (1,6)
Konsistente Strategie der Intervention (0–21)	21	18,4 (2,7)
*Subjektive Zielerreichung (0–15)*	10	11,0 (3,6)
Bewertung des betriebsärztlichen Erstgesprächs (Noten 1–6)	21	1,3 (0,7)
Bewertung des zweitägigen Teilhabe-Assessment (Noten 1–6)	21	1,9 (0,9)
*Bewertung der betrieblichen Nachsorgegespräche (Noten 1–6)*	10	1,8 (1,6)

*M* Mittelwert, *SD* Standardabweichung

Nichtkursiv = im ersten Nachsorgegespräch erhoben, *kursiv* = im letzten Nachsorgegespräch erhoben

### Veränderungen von Gesundheit und Arbeitsfähigkeit

**Figure 1:**
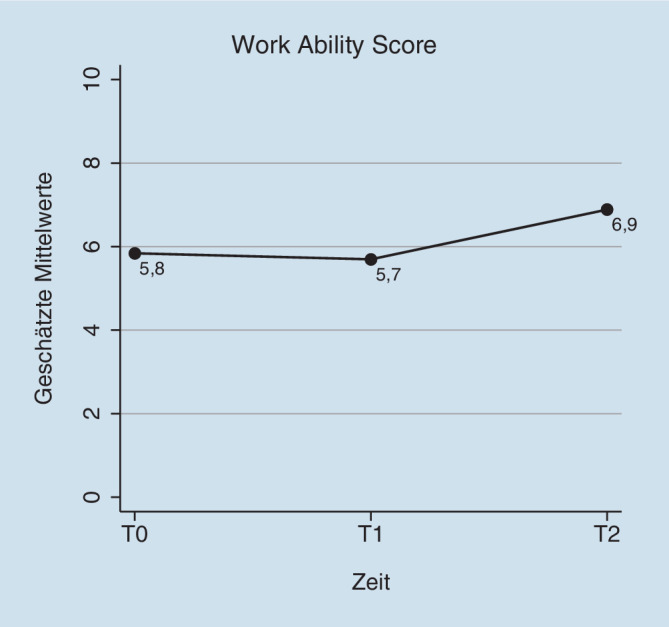

In Tab. 4 sind die Ergebnisse der gemischten Modelle dargestellt, mit denen die Veränderung von Gesundheit und Arbeitsfähigkeit im Beobachtungszeitraum geprüft wurde. Vom betriebsärztlichen Erstgespräch zum letzten Nachsorgegespräch berichteten die Teilnehmenden eine Verbesserung ihrer subjektiven Arbeitsfähigkeit um durchschnittlich 1,1 Punkte (Differenz = 1,05; 95 % KI −0,28 bis 2,37; d = 0,48) und eine starke Veränderung ihrer allgemeinen Gesundheit um 1,5 Punkte (Differenz = 1,52; 95 % KI 0,37–2,67; d = 0,97); (Abb. 1 und 2). Die körperliche Funktionsfähigkeit verbesserte sich im Beobachtungszeitraum um 3,7 Punkte (Differenz = −3,65; 95 % KI −6,33 bis −0,96; d = −0,64). Hinsichtlich Arbeitsbelastungen und Arbeitsbedingungen zeigte sich in der Skala Arbeitsplatzzufriedenheit eine moderate Verbesserung (Differenz = 17,2; 95 % KI 5,19–29,22; d = 0,72). Zudem berichteten die Teilnehmenden im Beobachtungszeitraum eine leicht verringerte Arbeitsunfähigkeit (Differenz in Wochen) während der vorangegangen 6 Monate (Differenz = −1,06; 95 % KI −2,90 bis 0,77; d = −0,19).

**Table 4:** 

Zielkriterien Messzeitpunkt	GM (SF)	Differenz T1–T0 T2–T0	95 % KI	Effektstärke Cohen’s d
*Arbeitsfähigkeit (Work-Ability-Score 0–10)*				
Erstgespräch (T0)	5,84 (0,45)	–	–	–
Erstes Nachsorgegespräch (T1)	5,70 (0,49)	−0,14	−1,15; 0,86	−0,07
Letztes Nachsorgegespräch (T2)	6,89 (0,66)	1,05	−0,28; 2,37	0,48
*Allgemeine Gesundheit (0–10)*				
Erstgespräch	5,24 (0,36)	–	–	–
Erstes Nachsorgegespräch	5,60 (0,39)	0,36	−0,53; 1,25	0,23
Letztes Nachsorgegespräch	6,76 (0,54)	1,52	0,37; 2,67	0,97
*Angst (0–6)*				
Erstgespräch	2,03 (0,33)	–	–	–
Erstes Nachsorgegespräch	1,90 (0,35)	−0,13	−0,71; 0,45	−0,07
Letztes Nachsorgegespräch	1,92 (0,44)	−0,11	−0,87; 0,66	−0,06
*Depressivität (0–6)*				
Erstgespräch	2,05 (0,30)	–	–	–
Erstes Nachsorgegespräch	1,92 (0,33)	−0,14	−0,88; 0,60	−0,10
Letztes Nachsorgegespräch	1,69 (0,46)	−0,37	−1,34; 0,60	−0,28
*Körperliche Funktionsfähigkeit (0–24)*				
Erstgespräch	7,69 (1,07)	–	–	–
Letztes Nachsorgegespräch	4,04 (1,49)	−3,65	−6,33; −0,96	−0,64
*Physische Arbeitsbelastung (0–15)*				
Erstgespräch	7,23 (0,92)	–	–	–
Letztes Nachsorgegespräch	9,00 (1,18)	1,77	−0,10; 3,64	0,38
*Psychische Arbeitsbelastung (0–100)*				
Erstgespräch	54,3 (2,74)	–	–	–
Letztes Nachsorgegespräch	51,1 (4,24)	−3,25	−12,04; 5,54	−0,25
*Soziale Unterstützung im Beruf (0–100)*				
Erstgespräch	62,0 (5,24)	–	–	–
Letztes Nachsorgegespräch	69,4 (7,44)	7,42	−6,37; 21,22	−0,26
*Arbeitsatmosphäre*				
Erstgespräch	80,8 (3,27)	–	–	–
Letztes Nachsorgegespräch	76,5 (4,72)	−4,24	−13,21; 4,72	−0,24
*Jobunsicherheit (0–100)*				
Erstgespräch	28,8 (5,33)	–	–	–
Letztes Nachsorgegespräch	20,6 (6,71)	−8,29	−18,71; 2,14	−0,30
*Arbeitsplatzzufriedenheit (0–100)*				
Erstgespräch	63,0 (4,36)	–	–	–
Letztes Nachsorgegespräch	80,2 (6,32)	17,2	5,19; 29,22	0,72
*Arbeitsunfähigkeit der letzten 6 Monate in Wochen*				
Erstgespräch	4,58 (1,06)	–	–	–
Letztes Nachsorgegespräch	3,51 (1,29)	−1,06	−2,90; 0,77	−0,19

*GM* Geschätzte Mittelwerte, *SF* Standardfehler, *T0* Erstgespräch, *T1* erstes Nachsorgegespräch, *T2* letztes Nachsorgegespräch

**Figure 2:**
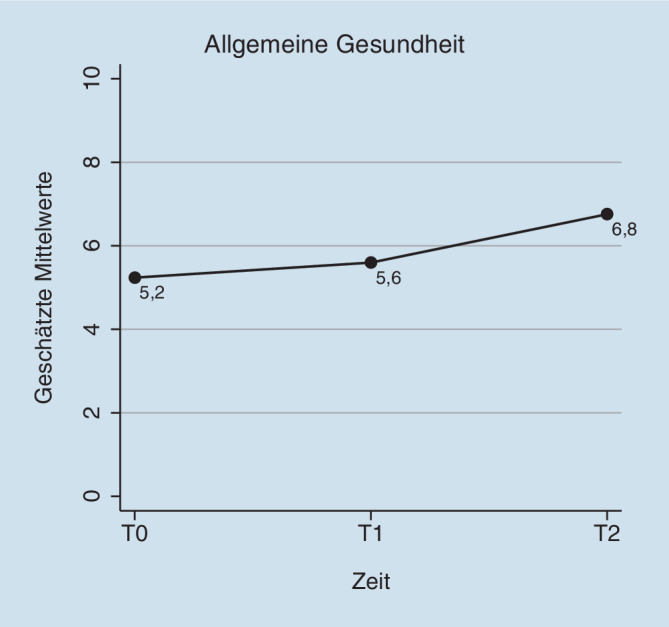

### Sensitivitätsanalyse

Die in der Sensitivitätsanalyse geschätzten Veränderungen zeigten eine Verbesserung der Teilnehmenden im Beobachtungszeitraum um durchschnittlich 1,7 Punkte (Differenz = 1,73; 95 % KI 0,35–3,11; d = 0,79) hinsichtlich ihrer subjektiven Arbeitsfähigkeit und um 1,7 Punkte (Differenz = 1,69; 95 % KI 0,83–2,55; d = 1,08) hinsichtlich der allgemeinen Gesundheit.

### Handlungsempfehlungen zur Sicherung beruflicher Teilhabe

Eine Übersicht von Handlungsempfehlungen, die als Ergebnis aus der zweitägigen ganzheitlichen Diagnostik abgeleitet wurden, zeigt Tab. 5. Die Empfehlungen konnten überwiegend den übergeordneten Bereichen Empfehlungen zur Lebensstiländerung, Anpassungen am Arbeitsplatz und der Arbeitsorganisation sowie ambulante Therapieleistungen zugeordnet werden.

**Table 5:** 

Code Family Zusammenfassung der Einzelcodes	Einzelcodes aus Abschlussberichten (*n* = 27) entwickelt
**Lebensstiländerungen** (k = 62 Empfehlungen)	Sport und Bewegung (k = 22), Erlernen von Entspannungstechniken (k = 16), Reduktion des Körpergewichts (k = 7), Ernährungsberatung (k = 6), Besuch einer Selbsthilfegruppe (k = 3), Verbesserung der Schlafhygiene (k = 3), Rückenschule (k = 3), Präventionsangebote der Krankenkassen (k = 2)
**Anpassungen am Arbeitsplatz und Arbeitsorganisation** (k = 31)	Verhältnisergonomie (k = 11), Verhaltensergonomie (k = 6), Anpassung der Arbeitsanforderungen (k = 4), Anpassung von Arbeitszeiten (k = 3), Abteilungswechsel (k = 2), innerbetriebliche Weiterbildung (k = 2), berufliche Orientierung (k = 1), Verbesserung des Betriebsklimas (k = 1), Verbesserung der Arbeitsatmosphäre (k = 1)
**Ambulante Therapieleistungen** (k = 30)	Ambulante Psychotherapie (k = 15), ambulante Physiotherapie (k = 12), ambulante Ergotherapie (k = 1), Traumatherapie (k = 1), Verhaltenstherapie (k = 1)

k = Anzahl der Empfehlungen

## Diskussion

Für die Machbarkeitsstudie, die die Entwicklung und Erprobung einer frühzeitigen diagnostischen Klärung von Interventionsbedarf begleitete, wurden 27 Mitarbeiter*innen mit gefährdeter beruflicher Teilhabe von den teilnehmenden Betriebsärzt*innen in Schleswig-Holstein, Hamburg und Mecklenburg-Vorpommern rekrutiert.

Ein Ziel der Machbarkeitsstudie war die Implementierung des Rekrutierungszugangs bei beteiligten Betriebsärzt*innen und den von ihnen betreuten Betrieben. Ein wichtiger Indikator für eine erfolgreiche Implementierung ist die Erreichung der intendierten Zielgruppe, für die die Intervention entwickelt wurde [18], da Abweichungen die Wirksamkeit der Intervention reduzieren können [37]. Es zeigte sich, dass die Betriebsärzt*innen die für das Modellvorhaben intendierte Zielgruppe rekrutierten. Die Teilnehmenden berichteten überwiegend eine mäßige bis kritische subjektive Arbeitsfähigkeit und einen vergleichsweise eingeschränkten Gesundheitszustand [38]. Im Vergleich zu einer Kohortenstudie, in der Erwerbstätige im Alter von 45 bis 59 Jahren in Deutschland rekrutiert wurden, berichteten die Teilnehmenden von GIBI in der Ersterhebung eine durchschnittlich um einen Punkt geringere subjektive Arbeitsfähigkeit (Work-Ability-Score) sowie allgemeine Gesundheit (COPSOQ; [39]). Die Stichprobe der vorliegenden Studie weist mit rund zwei Dritteln einen höheren Anteil an weiblichen Teilnehmenden auf. Ein Grund dafür könnte die Erwerbstätigenstruktur der teilnehmenden Unternehmen sein (z. B. Krankenhaus und Betreuungseinrichtungen). Zugleich zeigt sich in Studien bei Frauen eine insgesamt höhere Inanspruchnahme von Angeboten der betrieblichen und individuellen Gesundheitsförderung [8, 40, 41].

Zudem beeinflusst der Kontext die Durchführung und Implementierung von komplexen Interventionen [42]. Die Mehrheit der Teilnehmenden in unserer Studie arbeitet in Berufen mit hohen fachlichen und komplexen Anforderungen. Die Betriebsärzt*innen haben somit eher Fachkräfte rekrutiert, die Berufe ausüben, die komplexere Kenntnisse und Fertigkeiten erfordern und die, in Anbetracht des Fachkräftemangels, bei Arbeitsunfähigkeit besonders schwer zu ersetzen sind. Die Implementierung unserer komplexen Intervention spricht also womöglich besonders Unternehmen und Arbeitgeber an, die einen hohen Bedarf an Fachkräften haben.

Neben der erreichten Zielgruppe ist die Umsetzung der Interventionsinhalte ein wichtiges Merkmal zur Bewertung der Implementierung von Interventionen [18]. Studien zeigen, dass der persönliche Kontakt mit Betriebsärzt*innen die Wiedereingliederung unterstützt. Zudem erleichtert die gemeinsame Identifizierung von Barrieren und unterstützenden Faktoren am Arbeitsplatz die Rückkehr in Arbeit und reduziert Fehlzeiten [13, 43]. Die Teilnehmenden bewerteten die Interventionsbausteine und die Zusammenarbeit mit ihren Betriebsärzt*innen mit gut bis sehr gut. Die Inhalte der Interventionsbausteine wurden wie geplant umgesetzt. Dies zeigen die hohen Werte zur erhaltenen Therapiedosis und zur konsistenten Strategie der Intervention aus Sicht der Teilnehmenden. Entscheidend dafür war die gemeinsame Entwicklung der Intervention und die Durchführung von Schulungen aller beteiligter Personen zur Durchführung der Interventionselemente von GIBI. Weiterhin berichteten die Teilnehmenden hohe Werte in der interventionsspezifischen subjektiven Zielerreichung, die als Indikator für die Qualität der Intervention steht [18]. Zusammengefasst ermöglichte uns die gemeinsame Entwicklung der Interventionsinhalte und Durchführungsmaterialien sowie der enge Kontakt zu den Betriebsärzt*innen Umsetzungsprobleme aufzudecken und diese für die nachfolgende randomisierte kontrollierte Studie zu berücksichtigen [17].

Die beobachteten Veränderungen im Beobachtungszeitraum von Gesundheit, Arbeitsfähigkeit, körperlicher Funktionsfähigkeit und Arbeitsplatzzufriedenheit waren relevant und sind in Einklang mit den Ergebnissen der Studie GABI (Grundfos-Aukrug zur Erhaltung der Beruflichen Integration; [9]). Bei GABI wurde ein ähnlicher Ansatz erfolgreich getestet und für alle Teilnehmenden die Arbeitsfähigkeit stabilisiert oder verbessert [9].

Durch die standardisierte Dokumentation der Ergebnisse der zweitägigen Diagnostik wurden aus GIBI abgeleitete Handlungsempfehlungen zusammengefasst, die wie bei GABI unterschiedlichen Oberkategorien zugeordnet werden können [9]. Für die Ableitung konkreter Handlungsempfehlungen war die psychosomatische Diagnostik zentral, die in vielen Fällen wesentlich zur Klärung der zugrundeliegenden gesundheitlichen Problematik beigetragen hat.

Bei der kritischen Einordung der Ergebnisse sind eine Reihe von Limitationen zu beachten. Erstens war die Stichprobe sehr klein, und es konnte nicht die geplante Stichprobengröße von 30 Personen zur Erprobung der Intervention erreicht werden. Ein Grund war, dass einige Betriebe die Teilnahme an GIBI (vorerst) ablehnten, da die für die Betriebe zuständigen Betriebsärzt*innen mit der Einbindung in die Test- und Impfstrategie gegen COVID-19 teilweise komplett ausgelastet waren. Zudem könnte auch die betriebsärztliche Struktur die Implementierung der Intervention sowie Rekrutierung beeinflusst haben [42]. Die Mehrheit der Betriebsärzt*innen, die für die Erprobungsphase rekrutierten, war angestellt. Zwar konnten wir auch Betriebsärzt*innen schulen, die Teil von betriebsärztlichen Diensten waren. Diese starteten jedoch zum Zeitpunkt der Erprobungsphase noch nicht mit GIBI. Mögliche Selektionseffekte in der Rekrutierung können daher nicht ausgeschlossen werden. Insgesamt reichte die Stichprobengröße dennoch aus, um die erbrachte und wahrgenommene Therapiedosis zu erfassen und um statistisch signifikante Veränderungen von Gesundheit und Arbeitsfähigkeit abzubilden. Zweitens war der Rücklauf zur Zweit- und Drittbefragung sehr gering. Dies war teilweise auf den Wechsel von Betriebsärzt*innen innerhalb der teilnehmenden Betriebe und Zuständigkeiten zurückführbar. Im Rahmen der anschließenden Hauptstudie zur Bewertung der Wirksamkeit der Intervention werden die Nachbefragungsunterlagen postalisch an die Teilnehmenden versandt. Drittens wurden die Teilnehmenden direkt am Ende der Intervention zuletzt befragt. Eine längere Nachbeobachtung mit einem deutlichen zeitlichen Abstand nach Abschluss der Intervention könnte zeigen, ob die gesundheitlichen Veränderungen nachhaltig sind. Viertens wurden keine detaillierten Angaben zu den Inhalten und zur Dauer der betrieblichen Nachsorgegespräche erfasst. Für die anschließende randomisierte kontrollierte Studie wurde daher ein digitales Dokumentationstool für die Betriebsärzt*innen erstellt, um die Dauer, Umsetzung und Inhalte standardisiert zu erfassen. Fünftens haben wir ein Prä-Post-Design gewählt, um die Entwicklung, Implementierung und Erprobung der Intervention zu begleiten. Das gewählte Studiendesign lässt keine kausalen Schlussfolgerungen für die Veränderungen von Gesundheit und Teilhabe zu. Zur Bewertung der Wirksamkeit des neuen Versorgungsangebots wird aktuell eine randomisierte kontrollierte Studie mit Wartekontrollgruppe durchgeführt (DRKS00027577; [17]). Die randomisierte Zuordnung zu Interventions- und Kontrollgruppe gewährleistet, dass mögliche Unterschiede bei der Nachbefragung kausal auf die Intervention zurückgeführt werden können. Insgesamt sollen 210 Personen an der randomisierten kontrollierten Studie teilnehmen. Für die vergleichende Prüfung der Wirksamkeit der Intervention werden Fragebogendaten zu 2 Erhebungszeitpunkten erhoben. Die Erstbefragung findet zum Studieneinschluss statt, die Nachbefragung erfolgt 6 Monate nach der Erstbefragung. Die primäre Zielgröße in den Analysen ist der Work-Ability-Score [22].

Diesen Limitationen stehen folgende Stärken gegenüber. Erstens ermöglichten die gemeinsam entwickelten Leitfäden zur Durchführung der betriebsärztlichen Elemente der Intervention sowie die gemeinsame Definition der zweitägigen Diagnostik in Entwicklungsworkshops eine vergleichbare Umsetzung der Intervention durch die Betriebsärzt*innen und Rehabilitationseinrichtungen. Zweitens konnten durch die Implementierung der Intervention in drei großen Modellregionen die Zusammenarbeit zwischen Betriebs- und Rehabilitationsmedizin gestärkt werden und unterschiedliche regionalspezifische Strukturen der betriebsärztlichen Betreuung und Unternehmensdichte berücksichtigt werden. Drittens hatten die Studienkoordinator*innen eine zentrale Bindegliedfunktion zwischen den Rehabilitationseinrichtungen sowie teilnehmenden Betrieben und Betriebsärzt*innen. Durch den regelmäßigen Austausch konnten Umsetzungsprobleme kommuniziert und gemeinsam Lösungen entwickelt werden, die unmittelbar an die beteiligten Akteure übermittelt wurden.

Zusammengefasst zeigt die Erprobungsstudie des Modellvorhabens GIBI erstens, dass durch die Betriebsärzt*innen die intendierte Zielgruppe erreicht wurde. Zweitens wurde die Intervention erfolgreich in drei Rehabilitationseinrichtungen implementiert und die definierten Inhalte der Maßnahme wurden umgesetzt. Drittens konnten erste Hinweise darüber erlangt werden, dass die Intervention zu einer relevanten Verbesserung von Gesundheit und Arbeitszufriedenheit führen kann.

## Fazit für die Praxis

- Betriebsärzt*innen haben eine wichtige Schlüsselfunktion bei der Initiierung und Begleitung von Rehabilitationsprozessen und können den Prozess der Rückkehr in Arbeit direkt am Arbeitsplatz unterstützen.
- Das Modellvorhaben GIBI verknüpft innerbetriebliche Kenntnisse und Kompetenzen der Betriebsmedizin mit dem Potenzial zur ganzheitlichen Diagnostik in Rehabilitationseinrichtungen und stärkt die interdisziplinäre Zusammenarbeit.
- Das Modellvorhaben GIBI bietet einen niedrigschwelligen Zugang zu einem vertrauensvollen, ganzheitlichen und arbeitsplatzorientierten Angebot, das zur Verbesserung von Gesundheit und Arbeitsfähigkeit führen kann.
- Die Ableitung passgenauer Handlungsempfehlungen zur Sicherung von Arbeitsfähigkeit bedarf einer ganzheitlichen und arbeitsplatzorientierten Diagnostik.
